# Association of *KRAS* G12C Status with Age at Onset of Metastatic Colorectal Cancer

**DOI:** 10.3390/cimb46020088

**Published:** 2024-02-04

**Authors:** Marcelo Sunagua Aruquipa, Renata D’Alpino Peixoto, Alexandre Jacome, Fernanda Cesar, Vinicius Lorandi, Rodrigo Dienstmann

**Affiliations:** 1 Oncoclinicas Gastrointestinal Oncology Department, São Paulo 04538-132, SP, Brazil; marceloporfiriosunaguaaruquipa@gmail.com; 2 Oncoclinicas Gastrointestinal Oncology Department, Belo Horizonte 34006-059, MG, Brazil; alexandre.jacome@medicos.oncoclinicas.com; 3 Oncoclinicas Gastrointestinal Oncology Department, Vitoria 29050-400, ES, Brazil; fernanda.cesar@medicos.oncoclinicas.com; 4 Oncoclinicas Gastrointestinal Oncology Deparment, Porto Alegre 90610-001, RS, Brazil; vinicius.lorandi@medicos.oncoclinicas.com; 5 Oncoclinicas Precision Medicine, São Paulo 04513-020, SP, Brazil; rodrigo.dienstmann@oncoclinicas.com

**Keywords:** colorectal cancer, *KRAS* G12C, *BRAF*, microsatellite instability

## Abstract

The association of age at the onset of CRC and the prevalence of a *KRAS* G12C mutation is unclear. A retrospective, multicenter study evaluating metastatic CRC patients from January 2019 to July 2023, treated at the Oncoclinicas units and tested for tissue based *KRAS*/*NRAS* and *BRAF* mutations in a centralized genomics lab. A mismatch repair (MMR) status was retrieved from different labs and electronic medical records, as were patient demographics (age, gender) and tumor sidedness. The chi-square test was used to examine the association between clinical and molecular variables, with *p* value < 0.05 being statistically significant. A total of 858 cases were included. The median age was 63.7 years (range 22–95) and 17.4% were less than 50 years old at the diagnosis of metastatic CRC. Male patients represented 50.3% of the population. The sidedness distribution was as follows: left side 59.2%, right side 36.8% and not specified 4%. The prevalence of the *KRAS* mutation was 49.4% and the *NRAS* mutation was 3.9%. Among *KRAS* mutated tumors, the most common variants were G12V (27.6%) and G12D (23.5%), while *KRAS* G12C was less frequent (6.4%), which represented 3.1% of the overall population. The *BRAF* mutant cases were 7.3% and most commonly V600E. Only five (<1%) non-V600E mutations were detected. MSI-high or dMMR was present in 14 cases (1.6%). In the age-stratified analysis, left-sidedness (*p* < 0.001) and a KRAS G12C mutation (*p* = 0.046) were associated with a younger age (<50 years). In the sidedness-stratified analysis, a *BRAF* mutation (*p* = 0.001) and MSI-high/dMMR status (*p* = 0.009) were more common in right-sided tumors. Our data suggest that *KRAS* G12C mutations are more frequent in early-onset metastatic CRC. To the best of our knowledge, this is the largest cohort in the Latin American population with metastatic CRC reporting *RAS*, *BRAF* and MSI/MMR status.

## 1. Introduction

Colorectal cancer (CRC) is the third most common neoplasm diagnosed worldwide in men and women and it is second in terms of cancer related mortality [1]. In Brazil, CRC incidence varies according to the region, oscillating from the second to the fourth most frequent diagnosed malignancy, according to data published from the Brazilian National Cancer Institute (INCA) [2]. The incidence of early onset CRC (EOCRC), which occurs in persons <50 years old, has been rising in recent years, especially in high-income countries. This increase can be explained by the exposure to risk factors since childhood and adolescence, such as sedentarism, an industrialized diet and intestinal microbiota alterations [3,4]. Despite the fact that the treatment options for EOCRC patients and patients above 50 years are the same, depending on the choice of performance and not on the age, a survival analysis using 50 years as the cutoff surprisingly revealed there were no statistical differences in the survival between patients younger and older than 50 years [5]. Nonetheless, according to the Global Cancer Observatory (GLOBOCAN) estimates, low and middle income countries will also have an increment EOCRC incidence in the next 20 years because of the increasing availability of diagnostic tools in these regions, like colonoscopy and imaging [6].

Some medical societies have suggested a reduction in the age of initiating CRC screening from 50 to 45 years [7]. Interestingly, a meta-analysis including 51,811 individuals who underwent colonoscopy in four continents showed that the average risk population aged 45 to 49 years had similar rates of CRC in comparison to the individuals aged 50 to 59 years, suggesting that expanding screening to those aged 45 to 49 years could be beneficial [8].

Precision oncology is a field in constant evolution, focusing on tailoring cancer therapies according to specific genetic alterations from the patient’s tumor with the aim of obtaining better clinical outcomes [9]. Although fluoropyrimidine-based chemotherapy remains the backbone of systemic therapy for metastatic colorectal cancer (mCRC), a cancer that has spread outside the colon to distant organs. An adequate characterization of the tumor molecular profile in addition to tumor sidedness has an important role in refining the adequate combination and sequence of therapies. The inhibition of the epidermal growth factor receptor (EGFR) offers more benefit in left-side *RAS* wild-type patients, while antiangiogenics drugs are better for *RAS*-mutant patients irrespective of side and in case of right-sided RAS wild-type tumors [10].

Likewise, several studies have shown that the *BRAF* V600 mutation is a powerful negative prognostic marker in mCRC, leading to the investigation of BRAF inhibitors combinations to improve the clinical outcomes [11]. Furthermore, high microsatellite instability (MSI-high) or defective mismatch repair (dMMR) have become meaningful prognostic and predictive biomarkers, since these tumors tend to portend better prognosis in early stages and tend to be highly sensitive to immune checkpoint inhibition in the metastatic setting [12].

According to various consensus, an adequate pathology report should contain data of *RAS* and *BRAF* mutation status as well as MSI/MMR [13]. In recent years, with the development of specific *KRAS* G12C inhibitors, this information is also being used for treatment decision making [14].

The access to oncology consultations and medical facilities is not equal throughout the Brazilian territory. Laboratories with the capacity to run molecular profiling in solid tumors are concentrated in large cities from the southeast region [15]. The coordination of a network of oncology units from diverse regions of the country to a centralized molecular pathology laboratory has increased access to reliable results [16]. Our study is the effort of our network to analyze the molecular profile of mCRC patients with standard-of-care biomarkers and integrate with patient demographics and tumor characteristics.

## 2. Materials and Methods

Population: A retrospective and multicenter study evaluating adult (>18 years-old) patients, all with stage IV metastatic CRC; the definition of metastatic disease included the presence of disease (proven by biopsy or imaging) in distant organs or membranes (example: pleura and peritoneum) also including non-intra-abdominal lymph nodes. The patients were treated at Oncoclinicas units in Brazil from January 2019 to July 2023 and were tested for *KRAS*/*NRAS* and *BRAF* mutations in a centralized genomics lab (Oncoclinicas Precision Medicine). Demographic and clinical data (gender, age, region of origin, and sidedness of the tumor) were retrieved from electronic medical records, together with the MSI/MMR status, if not performed in the central laboratory.

Analysis and Genotyping: The specimens came to our central laboratory review from several Brazilian centers within the Oncoclinicas network, from January 2021 to July 2023. The cases were reviewed by a group of expert pathologists, all of whom performed tissue-based analysis. The samples were obtained either from the primary tumor by colonoscopy or from biopsies of distant metastasis. No liquid biopsies were included. DNA was extracted from tissue samples (FFPE) using QIASymphony extractions kits and the appropriate tissue area was defined as 100–300 µ^2^ with >10% tumor cells. An input of 100 ng was required. The NGS library was prepared using a QIAseq Targeted DNA Custom Panel (QIAGEN, Hilden, Germany). Sequencing was performed in the Illumina platform (MiSeq, San Diego, CA, USA) in paired end 2 × 150 cycles. The test covered 23 cancer genes, with oncogenic mutations in *KRAS* (exons 2 to 5), *NRAS* (exons 2 to 4) and *BRAF* (exons 7, 11, 12, 15, 16) analyzed as part of a patient support program sponsored by Amgen (RAStrear, Thousand Oaks, CA, USA). Analytical sensitivity was defined at 1% variant allele frequency [17,18]. MSI or MMR status was examined using standard PCR-based techniques or immunohistochemistry (IHC) of MLH1, MSH2, MSH6 and PMS2 proteins, respectively [19].

Statistical analysis: Descriptive statistics were used to characterize demographic and clinical data. Frequencies and percentage were used for the categorical variables and mean, median, and standard deviation were used for numerical variables. The correlation among variables was conducted using the Chi-square and Fisher’s exact test when applicable. Because of the various types of *KRAS* mutations, the Bonferroni method for multiple testing was used for adjustment of the *KRAS* correlation *p* value. Age groups were categorized in three strata: <50 years (as the age of recommendation for screening colonoscopy), 50–80 years and >80 years. Right-sided tumors encompassed cecum to the transverse colon, and left-sided tumors from the splenic flexure to rectum. Statistical significance for all results was established as *p* value < 0.05. The statistical software used was SPSS version 24. The study was approved by the Institutional Review Board on 12 July 2023, protocol code 70914323.0.0000.0070.

## 3. Results

A total of 858 patients were included in our database (Table 1). Regarding demographic data, male patients represented 50.3%, the most frequent region of origin of the samples was the southeast region (76.1%), and the most frequent states were Rio de Janeiro (32.1%) and Minas Gerais (31.7%). The median age was 63.7 years (range 22–95 years). The age groups distribution of participants was: 149 (17.4%) <50 years, 602 (70.1%) from 50 to 80 years and 107 (12.5%) >80 years. Right-sided tumors were present in 316 (36.8%), left-sided tumors in 508 (59.2%), and 34 (4%) cases were not specified.

**Figure 1:**
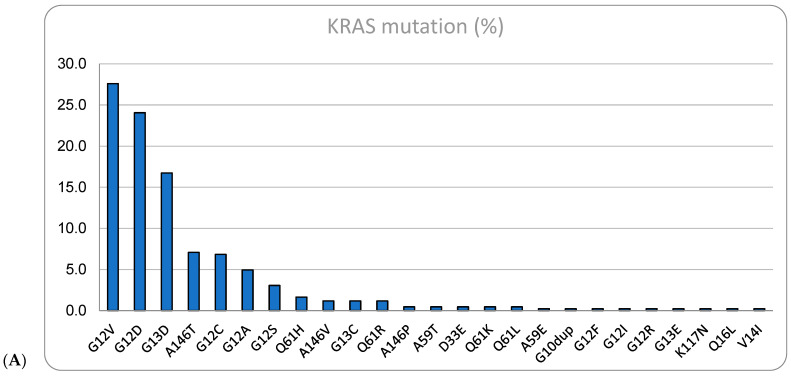

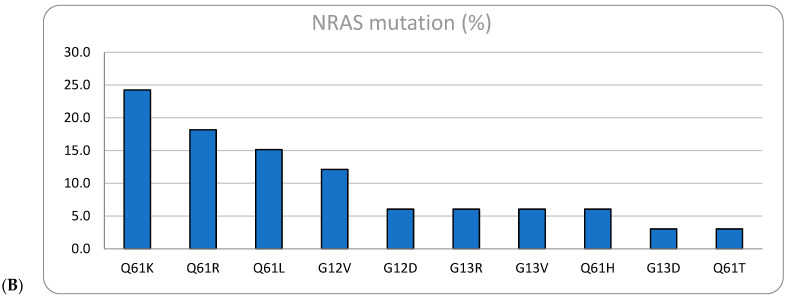
Distribution of (**A**) *KRAS* mutations and (**B**) *NRAS* mutations in samples of metastatic CRC patients from January 2019 to July 2023 treated at the Oncoclinicas network.

Of the 858 cases, 401 (46.7%) were RAS wild type, the proportion of *KRAS* and *NRAS* mutations was: 424 (49.4%) and 33 (3.9%), respectively. The most common *KRAS* mutations were G12V (27.6%), G12D (24.1%) and G13D (16.7%) (Figure 1A). Of special interest, the specific *KRAS* G12C mutation was present in 27 cases, representing 6.8% of the total *KRAS*-mutated patients and 3.1% of the total population. The most common *NRAS* mutation (Figure 1B) was the Q61K (24.2%). Only 63 cases (7.3%) were identified as *BRAF* mutant, most of them with the classical V600E, but there were five cases of non-canonical V600E mutation (D201N, D549N, D594G, G466V and V600K). MSI-high/dMMR was present in 14 cases (1.6%).

We performed two correlation analyses (using the chi-square method) stratifying by age (Table 2) and sidedness (Table 3). In the age-stratified analysis, the variables with statistical significance were sidedness (*p* < 0.001) and the *KRAS* G12C mutation (*p* = 0.046 by the chi-square method and *p* = 0.0018 by the Bonferroni multiple testing method, with an adjusted significance threshold of *p* < 0.0019). There was a higher incidence of left-sided tumors in patients < 50 years, while right-sided tumors were more frequent in the elderly population (>80 years). Likewise, in the sidedness-stratified analysis, age also resulted in a significant correlation (*p* < 0.001) (Table 3). Other variables that demonstrated statistical significance were *BRAF*-mutant (*p* = 0.001) and MSI-high/dMMR status (*p* = 0.009), which were more commonly found in right-sided tumors.

## 4. Discussion

To our knowledge, this is the first work to assess the mutational status (*KRAS*, *NRAS*, *BRAF*) together with the MSI/MMR status of metastatic CRC in the Brazilian population and the largest analysis in Latin America. Indeed, most previous studies have been unicentric or focused only on one molecular characteristic [20,21,22]. We considered young patients less than 50 years old according to the recommendation for CRC screening. In our data, 17.4% were <50 years old, with a predominance of left-sided tumors (76.8%). As an example, a study in Belo Horizonte, Brazil with 388 patients found that 20% of patients were younger than 50 years and 74% of the participants had left-sided tumors, but no correlation with any mutational status was encountered [20]

Our distribution of the RAS mutant status in total population (*KRAS* 49.4% and *NRAS* 3.9%) was similar to the 50.3% *KRAS* and 3.8% *NRAS*-mutant patients reported in a large Brazilian cohort of 2067 tissue samples, including both metastatic and localized disease. In the same study, the prevalence of a *BRAF*-mutant status was 6.6% as compared to 7.3% in our cohort. Additionally, the study found a significant correlation between *NRAS* and *BRAF*-mutant status with older age (>75 years old) [21].

Although no significant association between *KRAS*, *NRAS*, or *BRAF* status and age at onset of CRC was identified in our data, we did find a statistically significant correlation (*p* = 0.046) between the specific *KRAS* G12C mutation and patients younger than 50 years. The prevalence of this specific mutation in our entire population was 3.1% (*n* = 27), a lower frequency in comparison to a large Japanese database, which reported a prevalence of 6.5% (*n* = 45), but most of its population was older than 65 years. However, the study did not find a significant association of the *KRAS* G12C mutation with age (*p* = 0.879) or sidedness (*p* = 0.776) [23]. Similarly, in the phase I/II trial of adagrasib plus cetuximab in pre-treated *KRAS* G12C mutated mCRC patients, most patients were older than 60 years [24].

Regarding the Brazilian population, a multicentric study focusing only on *KRAS* status including 989 patients found a 38% prevalence of *KRAS*-mutant tumors, of whom G12D was the most common mutation [22]. Numerically, the largest multicentric study in the Brazilian population evaluated the prevalence of *KRAS* mutations in 8234 CRC samples and reported a prevalence of 31.9%, of which *KRAS* G12V was the most frequent, with most samples coming from the southeast region [25]. This fact was also seen in our data, with 76.1% of the samples also coming from the southeast region, a weakness of our study is that we had no representation from the north region of Brazil.

Similar to our data, an Argentinian study with 9150 CRC patients reported a frequency of *KRAS* and *NRAS* mutations of 43.4% and 3.6%, respectively. Furthermore, their *BRAF* mutation rate was 12.1%, slightly higher than in our data [26].

A large American database with 13,336 advanced CRC patients, reported a prevalence of 51.9% (*n* = 6926) of *KRAS* mutation, 4.5% (*n* = 594) of *NRAS* mutation, and 3.3% (*n* = 455) of MSI-high [27]. Whereas our MSI-high frequency was smaller when compared to this American study.

Unfortunately, only scarce data exist on the prevalence of MSI-high/dMMR in metastatic CRC patients in Brazil. A small unicentric study from Barretos, Brazil comprising both localized and metastatic CRC with only 95 patients, whose samples were analyzed with PCR techniques, found a prevalence of 13.3% (*n* = 12) of MSI-high patients [28]. However, these results can be biased since most of the patients had a localized disease, when the prevalence of MSI-high tumors is higher in early stages.

Moreover, the Keynote-177 trial reported an association of MSI-high/dMMR with sidedness, with 67% right-sided and 30% left-sided tumors [29]. Our results showed a similar proportion, with 14 patients having an MSI-high status, significantly associated with right-sided tumors (*p* = 0.019).

In our data, the prevalence of *BRAF* mutation was 7.3% with a significant association (*p* = 0.001) with right-sided (11%) in comparison to left-sided tumors (3%). Our finding compares similarly to an international meta-analysis including 15,981 patients with mCRC that reported a significant difference (*p* = 0.0001) in the *BRAF* mutant status between right (16.3%) and left-sided (4.3%) tumors [30].

Finally, we must emphasize the importance of a structured pathology network that provides access to molecular testing to a large population [31]. The cooperation between various oncology-dedicated centers has a key role in the development of research, prevention, and treatment strategies in colon cancer [16]. This is a one of the challenges in a large and underdeveloped country such as Brazil, as most of the medical facilities and technologies are concentrated in the southeast region, from where more than three quarters of our samples were derived [32].

Adequate information about the molecular status of mCRC patients has an impact on the clinical decision-making process for oncologists, as appointed by a nation-wide survey conducted by the Brazilian Group of Gastrointestinal Tumors [33]. In addition, the knowledge generated by our study could help health care authorities to develop a more efficient administration of resources in Brazil, as demonstrated in a cost-comparison analysis of sequential therapies in the Brazilian population [34].

Finally, for a long time *KRAS* G12C was a known but difficult to inhibit, oncogenic driver related to a poor prognosis in comparison to other *KRAS* mutations and *RAS* wild-type tumors, and with the recent development of specific inhibitors, the understanding of this biomarker can be useful to improve the outcomes of EOCRC patients that so far have similar results to the population of 50 years or older [35].

## 5. Conclusions

In our Brazilian cohort of mCRC patients, frequencies of *RAS* and *BRAF* mutations were in line with worldwide data. However, we found a lower than expected frequency of MSI-high/dMMR tumors. The *KRAS* G12C mutation was associated with early-onset mCRC, an emergent population in which KRAS G12C inhibitors might be useful. To the best of our knowledge, this is the largest cohort in the Brazilian population with mCRC reporting *RAS*, *BRAF,* and MSI-high status. Larger studies are needed to confirm these findings.

## Figures and Tables

**Table 1:** Characteristics of patients.

	Variables	Total (*n* = 858)	Percentage
Gender	Male	432	50.3%
	Female	426	49.7%
Region	Northeast	83	9.7%
	Midwest	32	3.7%
	Southeast	653	76.1%
	South	90	10.5%
State	Pernambuco	17	2%
	Paraiba	19	2.2%
	Sergipe	2	0.2%
	Bahia	45	5.2%
	Goais	6	0.7%
	Sao Paulo	75	8.7%
	Rio de Janeiro	275	32.1%
	Espiritu Santo	31	3.6%
	Minas Gerais	272	31.7%
	Parana	33	3.8%
	Rio Grande do Sul	51	5.9%
	Santa Catarina	7	0.8%
	Distrito Federal	25	2.9%
Age group	<50 years	149	17.4%
	50–80 years	602	70.1%
	>80 years	107	12.5%
Sidedness	Righ-side	316	36.8%
	Left-side	508	59.2%
	Not specified	33	3.9%
*RAS* status	*RAS* wild-type	401	46.7%
	*RAS* mutant	457	53.3%
	*KRAS* mutant	424	49.4%
	*NRAS* mutant	33	3.9%
	*KRAS* G12C mutant	27	3.1%
	*BRAF* mutant	63	7.3%
	MSI-high/dMMR	14	1.6%

Note: MSI: microsatellite instability, dMMR: deficient mismatch repair.

**Table 2:** Correlation between clinicopathological variables by age group.

Variables	<50 Years (*n* = 149)	50–80 Years (*n* = 602)	>80 Years (*n* = 107)	*p*-Value
Male	70 (47.0%)	307 (51.0%)	49 (45.8%)	*p* = 0.473
Female	79 (53.0%)	295 (49.0%)	58 (54.2%)	
Right-side	37 (29.5%)	223 (38.8%)	56 (52.8%)	*p* < 0.001
Left-side	106 (74.1%)	352 (61.2%)	50 (47.2%)	
RAS wild-type	75 (50.3%)	280 (46.5%)	46 (43.0%)	*p* = 0.733
*KRAS* mutant	68 (45.6%)	298 (49.5%)	58 (54.2%)	
*NRAS* mutant	6 (4.0%)	24 (4.0%)	3 (2.8%)	
*KRAS* G12C mutant	9 (13.2%)	17 (5.7%)	2 (3.4%)	*p* = 0.046
Other *KRAS* mutations	59 (86.8%)	281 (94.3%)	56 (96.6%)	
*BRAF* mutant	12 (8.1%)	42 (7.0%)	9 (8.4%)	*p* = 0.815
*BRAF* wild-type	137 (91.9%)	560 (93.0%)	98 (91.6%)	
MSI-high/dMMR	1 (0.7%)	10 (1.7%)	3 (2.8%)	*p* = 0.412
MSS/pMMR	307 (97.2%)	595 (98.3%)	104 (97.2%)	

Note: significant *p* < 0.05 values for sidedness and *KRAS* G12C status.

**Table 3:** Correlation between clinicopathological variables by sidedness.

Variables	Right-Side (*n* = 316)	Left-Side (*n* = 316)	*p* Value
Male	147 (46.5%)	261 (51.4%)	*p* = 0.175
Female	169 (53.5%)	247 (48.6%)	
<50 years	37 (11.7%)	106 (20.9%)	*p* < 0.001
50–80 Years	223 (70.6%)	352 (69.3%)	
>80 years	56 (17.7%)	50 (9.8%)	
RAS wild-type	153 (48.4%)	230 (45.3%)	*p* = 0.239
*KRAS* mutant	155 (49.1%)	254 (50.0%)	
*NRAS* mutant	8 (2.5%)	24 (4.7%)	
*KRAS* G12C mutant	10 (6.5%)	17 (6.7%)	*p* = 0.924
Other *KRAS* mutations	145 (93.5%)	237 (93.3%)	
*BRAF* mutant	35 (11.1%)	26 (5.1%)	*p* = 0.001
*BRAF* wild-type	281 (88.9%)	482 (94.9%)	
MSI-high/dMMR	9 (2.8%)	3 (0.6%)	*p* = 0.009
MSS/pMMR	307 (97.2%)	505 (99.4%)	

Note: significant *p* < 0.05 values for age, *BRAF* status, and MSI-high/dMMR status.

## Data Availability

Our database is not publicly available due to institutional privacy restrictions.
